# Cold Plasma‐Mediated Inactivation of Spore‐Forming Microorganisms: Mechanisms, Quality Attributes, and Efficiency Parameters

**DOI:** 10.1002/fsn3.70429

**Published:** 2025-06-18

**Authors:** Shiva Ezzati, Hossein Ahangari, Mohadeseh Mohammadian, Ali Khoshkalampour, Ehsan Moghaddas Kia, Zahra Ghasempour

**Affiliations:** ^1^ Department of Food Science and Technology, Faculty of Agriculture University of Tabriz Tabriz Iran; ^2^ Department of Food Science and Nutrition Maragheh University of Medical Sciences Maragheh Iran; ^3^ Department of Food Science and Technology, School of Nutrition Science and Food Technology Kermanshah University of Medical Science Kermanshah Iran; ^4^ Nutrition Research Center, Department of Food Science and Technology, Faculty of Nutrition and Food Sciences Tabriz University of Medical Sciences Tabriz Iran

**Keywords:** bacterial spore, cold plasma, inactivation mechanism, process variables

## Abstract

Bacterial spores are a dormant and non‐replicating state of bacteria, characterized by multiple layers of proteins, carbohydrates, and lipids. Spores from *Bacillus* and *Clostridium* species serve as main indicators of food safety, presenting considerable risks to consumers health. Heat sterilization is an easy and efficient technique; however, it compromises the quality and nutritional value of food. Cold plasma (CP), a developing non‐thermal sterilization method, has demonstrated significant promise in food sterilization. CP offers a promising alternative, as it does not cause irreversible damage to sensory or nutritional attributes and is more cost‐effective than traditional heat methods. The research explored the effects of different process parameters, primarily electrode type (direct or indirect plasma exposure), plasma power, duration of treatment, plasma‐induced dosage, distance of samples between electrodes, and the plasma gas on the bacteriostatic effectiveness. Additionally, the interaction between plasma‐induced reactive species and spore inactivation, as well as deterioration mechanisms, will be discussed thoroughly. CP can be utilized effectively, either independently or in combination with other methods, to enhance the efficacy of preservation techniques, thereby holding the potential to revolutionize food preservation practices significantly. Finally, a deeper insight into the relationship between spores and cold plasma is crucial for enhancing and improving cold plasma technology in the food industry.

## Graphical abstract

This review seeks to systematically assess the efficacy of cold plasma treatment in spore inactivation, elucidating its operational mechanisms through a comprehensive analysis of scientific literature that investigates the impact of various influencing factors. Additionally, it provides an overview of the characteristics of spore‐forming bacteria, the importance of food matrices, and the deactivation mechanisms facilitated by plasma.

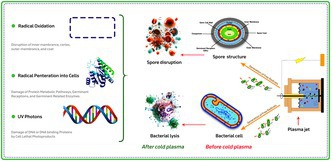

## 1 Introduction

The investigation of bacterial endospores began over 130 years ago, with pivotal contributions by Cohn and Koch in 1876. Despite extensive research into spore dormancy and resistance mechanisms, fundamental questions regarding heat inactivation remain unanswered. Early studies recognized the remarkable dormancy and durability of spores, and while significant advancements have been made, the underlying mechanisms are still not fully understood. Evidence suggests that specific components of spores, such as small acid‐soluble proteins, calcium, dipicolinic acid in the core, and peptidoglycan in the cortex, play critical roles in their resistance. Additionally, physical factors like core dehydration, which may induce a high‐viscosity or glassy state, have contributed to understanding spores' complex resistance properties. The initial categorization of spore‐formers, based on morphology in the late 19th century, laid the groundwork for classification systems that were later refined in the mid‐20th century before the advent of contemporary genetic methods (Zhu et al. 2022).

Food contamination by pathogens, molds, yeasts, and even spores can cause spoilage in which food materials undergo undesirable physico‐chemical and sensory changes (Langroodi et al. 2018; Mehdizadeh et al. 2018; Dadkhodazade et al. 2021). Spore contamination in food systems is a dynamic process influenced by spore characteristics, food matrix complexity, environmental conditions, and detection methods. Among these factors, storage temperature is key in maintaining spores' dormant state in food (Du et al. 2023; Aho‐Laukkanen et al. 2024). Soil concentrations of *Bacillus cereus* and *Clostridium* spp. can reach 10^5^–10^6^ spores per gram, directly threatening food products. Spore‐related infections, particularly those caused by *Clostridium* spp., are associated with significant morbidity, mortality, and financial costs. For example, *Clostridium difficile* infections alone account for an estimated $5.4 billion annually in the United States, with $4.7 billion attributed to healthcare facilities and $725 million to community settings. In untreated elderly patients, pseudomembranous colitis has a morbidity and mortality rate of 10%–20%. These statistics highlight the critical role of spores in food contamination and the pressing need for effective spore‐deactivation strategies (Sahra et al. 2021).

The high incidence of spore contamination in bulk and packaged food products, such as canned goods, poses significant health risks to consumers and represents a primary food safety concern requiring detailed evaluation. While effective for spore inactivation, traditional high‐heat treatments often degrade a food's nutritional and sensory quality. Increasing consumer demand for minimally processed foods with high nutritional value has driven research into advanced, non‐thermal technologies that preserve organoleptic properties while effectively inactivating spores. Non‐thermal methods such as gamma radiation, sonication, ultraviolet light, high‐pressure processing, and cold plasma have gained significant attention.

Cold plasma, an innovative and non‐thermal technology, has emerged as a promising sterilization technique in the food industry (Ozen et al. 2022). The first dielectric barrier discharge (DBD) apparatus was developed by Siemens in 1850 for ozone generation and water purification. Later, in 1928, Langmuir introduced the term “plasma” to describe oscillations in ionized gases (Bermudez‐Aguirre 2020). Plasma, regarded as the fourth state of matter, is formed by applying significant energy, resulting in ionization. The process generates chemically active species, including free electrons, radicals, positive and negative ions, ultraviolet photons, and excited or neutral atoms.

Recent applications of cold plasma include preserving juices, grains, meats, and dairy products while effectively inactivating spores within these foods. The germicidal activity of cold plasma is primarily attributed to the generation of active chemical species (Huang et al. 2023). The efficiency of cold plasma for spore and microbial inactivation is influenced by power, frequency, voltage, temperature, gas composition, and treatment time. Additionally, food matrix composition and moisture content significantly affect inactivation outcomes. Research has shown that different microorganisms exhibit variable sensitivities to plasma, with spores more resistant than vegetative cells. This underscores the need for comprehensive studies to better understand the interactions between cold plasma and target microorganisms (Valdez‐Narváez et al. 2024).

Advancing knowledge of technologies like cold plasma can assist the food industry in mitigating spore contamination while maintaining food safety. Since plasma technology relies solely on electrical energy, it represents a cost‐effective and sustainable alternative to conventional heat treatments, which typically depend on fossil fuels. Despite its potential, there is a lack of comprehensive reviews focused on applying cold plasma technology to inactivate foodborne spores from *Bacillus* spp. and *Clostridium* spp. This review seeks to address this gap by examining the use of cold plasma for spore deactivation, emphasizing its efficacy and potential to improve the management of spore contamination in food products.

## 2 Effective Factors in Cold Plasma Efficacy

### 2.1 Plasma Type

Advancements in plasma engineering have facilitated the development of various plasma sources capable of operating at atmospheric pressure. These sources are generally categorized into DBD, gliding arc discharges (GAD), corona discharges, glow discharges, radiofrequency plasma, and atmospheric pressure plasma jet (APPJ). DBD and APPJ are the food industry's most widely used and practical systems (Liu et al. 2023).

DBD plasma is generated between two metallic electrodes covered with a dielectric material, such as quartz, plastic, or ceramic. The discharge gap ranges from 100 μm to several centimeters. The dielectric layer prevents arc formation, which could produce a hot plasma, ensuring the temperature of neutral particles and ions remains relatively low. DBD is produced using high‐voltage pulses at frequencies ranging from 10 Hz to several kHz, and the concise duration of the discharges facilitates non‐thermal plasma generation (Sakudo et al. 2020).

Corona discharge occurs when high voltage is applied to sharp electrodes, such as tips, pinpoints, or thin wires, creating a high‐intensity electric field near these points. This electric field initiates the active zone of the corona and generates plasma. GAD is produced in a reactor with diverging metal electrodes operating at a potential difference of 9 kV and a current of 100 mA in outdoor conditions. A humid air inlet is introduced into the discharge gap, forming an arc in the narrowest inter‐electrode region. The inlet gas displaces the arc into the diverging area, allowing GAD to operate at high power levels under non‐thermal plasma conditions. GAD enhances plasma application efficiency and provides elevated power levels compared to other discharge types (Umair et al. 2022; Ma et al. 2022).

Glow discharge is a stable plasma phenomenon occurring at low gas pressures (1–1000 Pa) when a direct voltage between 100 V and several kilovolts is applied. In this process, electron energy exceeds that of ions and neutral gases, resulting in a non‐thermal, non‐equilibrium plasma. Glow discharge exhibits high spatial uniformity and can operate at low temperatures across large volumes, making it suitable for diverse applications (Sakudo et al. 2020).

Radiofrequency plasma is generated by introducing gas into an oscillating electromagnetic field created by an induction coil or external electrodes outside the reactor. This plasma type utilizes well‐established technology and operates at frequencies ranging from Hz to MHz, offering reliable and efficient plasma generation (Umair et al. 2022).

Plasma jet systems are distinct discharge types that combine features of other plasma configurations. The discharge's active region is influenced by a flowing auxiliary gas, which transports reactive particles from the electrode zone via ionization wave propagation, creating a small jet. Plasma jets generate streams of reactive particles and are commonly used in configurations such as plasma torches, jets, pens, and needles. Due to their simple design, ease of maintenance, and ability to operate at atmospheric pressure, plasma jets are widely applied in food treatment processes (Ma et al. 2022).

### 2.2 Influence of Treatment Matrices

Cold plasma achieves microbial inactivation in food primarily through the generation of reactive chemical species, such as reactive oxygen and nitrogen species, along with the emission of ultraviolet radiation and the application of electric fields. Extensive research has explored how treatment matrices affect the efficiency of cold plasma processing. For example, *Bacillus atrophaeus* vegetative cells and endospores were assessed within model systems comprising wheat, barley, and distilled water before being applied to a rubber substrate. Findings indicated that microbial inactivation was notably more difficult on the rubber surface than in liquid media primarily due to the robust adhesion of cells to the rubber material. Additionally, the wheat and barley matrices appeared to provide a protective effect against reactive plasma species, likely attributable to the inherent shielding properties of these matrices (Prakash et al. 2023).

In another investigation involving whole black peppercorns inoculated with spores and vegetative cells, inactivation was particularly challenging in surface regions characterized by cracks, grooves, and pits. This difficulty was mainly linked to shadowing effects, which obstructed the penetration of UV photons—commonly lethal to microbial cells and spores—into the complex microstructures of the peppercorn surface. Consequently, these structural features significantly diminished the antimicrobial efficacy of cold plasma treatment (Wang, Liu, et al. 2024).

In another research, cold plasma (CP), a promising nonthermal technology in food processing, was applied to blueberries for 0 to 120 s at a 7.5 cm distance, using a 4 cfm CP jet and 7 cfm ambient air. Microbial analysis showed that CP significantly (*p* < 0.05) reduced total aerobic plate counts and yeast/mold populations, with reductions of 0.8–1.6 log CFU/g immediately and 1.5–2.0 log CFU/g after 7 days. However, treatments longer than 60 s led to a decline in firmness partly due to mechanical damage. Anthocyanin content was significantly reduced at 90 s, and surface color parameters (L*, a*, and b*) were negatively affected, particularly after 120 s. Overall, CP shows strong potential for microbial decontamination of blueberries, provided that treatment conditions are properly optimized to minimize quality loss (Ji et al. 2020).

This study evaluated the effectiveness of a large‐gap atmospheric cold plasma (ACP) system using a high‐voltage dielectric barrier discharge (DBD) pilot‐scale reactor for decontaminating fresh produce while preserving quality. Both static and continuous ACP modes were tested on strawberries and spinach, showing significant reductions in bacterial populations. Static ACP treatment resulted in reductions of *Escherichia coli* and *Listeria innocua* by 2.0–2.2 and 1.3–1.7 log10 CFU/mL, respectively, while continuous treatment achieved a 3.8 log10 CFU/mL reduction for *L. innocua* on strawberries. No significant quality changes were observed in color, firmness, pH, or total soluble solids (TSS) between control and treated samples, with effects maintained throughout shelf‐life. The ACP treatment effectively controlled microbial contamination without negatively affecting the product's quality, making it a promising non‐thermal, water‐free technology for post‐package treatment (Ziuzina et al. 2020).

This study investigated the efficacy of low‐pressure cold plasma (LPCP), using oxygen as the process gas, for the inactivation of *Bacillus cereus* vegetative cells and spores in both inert and food‐based matrices. The results demonstrated significantly greater microbial reductions in vegetative cells compared to spores. The presence of a rice matrix exerted a protective effect, thereby diminishing the inactivation efficiency of spores. The inactivation kinetics were well described by the Weibull model, indicating a notable decline in microbial resistance with increasing treatment intensity. These findings highlight the crucial influence of food matrix composition on plasma effectiveness and support the potential application of LPCP as a non‐thermal pre‐treatment to enhance microbial safety in low‐water‐activity foods such as rice (Valdez‐Narváez et al. 2024).

The composition of food matrices significantly influences the efficacy of cold plasma treatments for microbial spore inactivation. High protein content can act as a protective barrier, as proteins may scavenge reactive species generated by cold plasma, thereby reducing its antimicrobial effectiveness. Conversely, certain carbohydrates can enhance spore germination, making spores more susceptible to plasma‐induced inactivation; however, some sugars may also react with reactive species, diminishing plasma efficacy. Fats and lipids tend to shield spores by absorbing reactive species and forming physical barriers, thus decreasing the overall inactivation efficiency of cold plasma treatments. Therefore, the specific composition of food products must be considered when applying cold plasma technology for microbial control (Bourke et al. 2017; Butscher et al. 2016).

### 2.3 Impact of Direct and Indirect Treatment

Depending on the interaction between plasma‐generated reactive species and the target surface, cold plasma treatments are broadly categorized into direct and indirect approaches. In indirect cold plasma systems, the food product is placed within a zone where reactive plasma species are present but remains outside the immediate discharge region of the plasma source. This configuration significantly restricts the interaction of reactive species with the food surface, thereby reducing the overall inactivation efficiency.

Conversely, the device delivers active species directly to the food surface in direct cold plasma systems, enabling direct contact between the plasma discharge and the target. This setup promotes more efficient interaction between reactive radicals and the surface, enhancing microbial inactivation. Several studies have investigated the efficacy of these two modes of treatment. For instance, bacterial biofilms formed by *E. coli* , *Bacillus*, and lactic acid bacteria (LAB) were exposed to direct and indirect plasma. The findings revealed that indirect plasma treatment was significantly less effective at disrupting biofilms (Prakash et al. 2023).

Similarly, the inactivation of *Bacillus atrophaeus* spores was evaluated under direct and indirect plasma conditions. The results demonstrated that direct plasma treatment exhibited markedly higher sporicidal activity than indirect exposure. The reduced efficacy of indirect treatment was attributed to two key factors: limited interaction between plasma‐generated reactive species and the spores and the rapid degradation of highly reactive transient species in the absence of direct contact. This degradation diminishes the probability of effective interaction with the spores, ultimately lowering the sporicidal efficiency (Wang, Yan, et al. 2024).

Depending on the interaction between plasma‐generated reactive species and the target surface, cold plasma treatments are broadly categorized into direct and indirect approaches. In indirect cold plasma systems, the food product is placed within a zone where reactive plasma species are present but outside the immediate plasma discharge region. This setup limits the penetration and concentration of highly reactive species, resulting in lower microbial inactivation efficiency. In contrast, direct cold plasma systems deliver reactive species directly to the food surface, where plasma discharge occurs in close proximity or direct contact with the target. This configuration enhances the interaction between the plasma's reactive species (such as reactive oxygen species [ROS] and reactive nitrogen species [RNS]) and microbial cells, thereby promoting more efficient microbial inactivation.

Several comparative studies have confirmed the superior efficacy of direct plasma. For instance, Prakash et al. (2023) reported that direct plasma treatment was significantly more effective than indirect plasma in disrupting biofilms formed by *E. coli* , *Bacillus*, and lactic acid bacteria (LAB). Similarly, Wang, Liu, et al. 2024, demonstrated that direct plasma exposure had substantially greater sporicidal activity against *Bacillus atrophaeus* spores compared to indirect exposure. The lower effectiveness of the indirect method was attributed to two main factors: (1) limited physical contact between reactive species and microbial targets and (2) the rapid degradation of short‐lived reactive species before reaching the surface.

In summary, direct plasma treatment is considered more effective for microbial inactivation due to its higher delivery of reactive species to the surface and stronger interaction with microbial structures. Furthermore, direct treatment typically generates a greater concentration of highly active radicals, as the discharge occurs in close proximity to the target, minimizing the loss of reactive species during transport. This makes direct plasma the preferred choice in applications requiring high antimicrobial efficacy.

This study systematically investigated the efficacy of direct and indirect cold atmospheric plasma (CAP) treatments in inactivating *Bacillus cereus* in black pepper. Direct‐CAP demonstrated superior inactivation, reducing vegetative cells by 5.1 log CFU/g after 10 min. Kinetic models were employed to optimize the inactivation process. Remarkably, CAP treatment preserved critical quality parameters such as color, flavor, and bioactive compounds (e.g., piperine and phenols), confirming its potential for industrial‐scale applications. These findings provide a theoretical foundation for CAP use in heat‐sensitive food matrices, offering a balanced approach to microbial safety and quality retention in spices (Wang, Liu, et al. 2024).

### 2.4 Impact of Voltage

In cold plasma research, microbial inactivation is closely linked to the intensity of the applied voltage. Reduced voltage levels decrease the production of electric sparks and ultraviolet (UV) radiation from the plasma electrode, thereby reducing the overall bactericidal efficiency. For instance, when the peak discharge voltage was lowered to 20 kV, a notable reduction in spark intensity resulted in diminished microorganism inactivation. No bactericidal effects were observed at even lower peak voltages of 18 and 16 kV. This lack of efficacy is attributed to the insufficient discharge energy, which fails to excite the surrounding gas, thereby preventing the ionization of reactive particles essential for microbial inactivation.

In contrast, higher voltage inputs significantly enhance gas ionization, generating more significant quantities of reactive species. These active species interact more effectively with microbial cells, improving inactivation efficiency significantly (Kulawik et al. 2023).

In the study on the effect of plasma on the inactivation of *Bacillus cereus* spores, the results showed that voltage and treatment time had a direct impact on reducing microbial load. Using plasma with 300 W power for 25 min achieved the highest reduction (> 5 log) in *Bacillus cereus* spores, indicating that higher voltage (and more power) has a stronger effect on spore inactivation. Regarding voltage sensitivity, plasma with oxygen gas showed greater effectiveness than air plasma due to the production of active oxygen radicals that cause oxidation and destruction of the spores. Moreover, increasing the voltage does not lead to more spore activation but rather directly kills them. Therefore, higher voltages are required for more effective microbial load reduction, but increasing voltage does not necessarily activate more strains (Teresa Fernández‐Felipe et al. 2024; Muratov et al. 2024).

### 2.5 Effect of the Working Gas

In this research, cold low‐pressure plasma (LPP) has emerged as an effective non‐thermal sterilization technique against spore‐forming bacteria, with its efficacy highly dependent on the type of working gas used. Oxygen and synthetic air were shown to produce the highest levels of reactive oxygen species (ROS), such as atomic oxygen and ozone, which play critical roles in damaging spore structures by oxidizing membrane lipids, degrading coat proteins, and inducing DNA strand breaks. This results in significantly higher inactivation rates compared to inert gases like argon. The study on *Bacillus subtilis* spores revealed that strains deficient in protective proteins (e.g., α/β‐type SASP), DNA repair enzymes (e.g., NHEJ pathway), and oxidative stress scavengers (e.g., catalase) were significantly more susceptible to LPP. These findings suggest that spore resistance is multifactorial and gas‐specific ROS production is a key mechanism of inactivation. Therefore, gases that produce higher levels of reactive species, particularly oxygen‐based ones, are more efficient for spore inactivation. Moreover, although increasing the voltage may enhance reactive species generation, its effectiveness plateaus if the resistance mechanisms are not overwhelmed, meaning that optimization requires balancing voltage, gas type, and exposure time (Muratov et al. 2024).

In this study, *Aspergillus flavus* was used as a model to compare the antifungal efficacy of atmospheric cold plasma (ACP) treatments in gas‐phase (GP) and plasma‐activated water (PAW) forms. Results showed that GP was more effective than PAW, achieving up to 2.2 log_10_ reductions in spore counts versus 0.6 log_10_ for the PAW. The inactivation efficiency was closely linked to the types and concentrations of reactive oxygen and nitrogen species (RONS), particularly long‐lived secondary species such as hydrogen peroxide, nitrate, and nitrite. Oxygen‐containing gases were inferred to produce higher levels of these active species, making them more effective against fungal spores. Mechanistically, PAW‐induced inactivation relied on acidification and chemical stress, while GP achieved superior results through direct oxidation, cell wall disruption, and structural damage from electric fields and reactive species. Overall, the type of working gas and delivery method critically influenced ACP efficacy, with gas‐phase plasma showing greater fungicidal performance due to its broader spectrum of reactive components and physical interactions (Los et al. 2020).

Studies have examined the effects of different carrier gas compositions on the inactivation of *B. atrophaeus* and *Bacillus subtilis* endospores. Pure argon demonstrated the highest inactivation efficiency, achieving reductions of 3.1 log CFU for *B. atrophaeus* and 2.4 log CFU for *B. subtilis* after 5 min of plasma exposure. A mixture of argon with 0.135% oxygen and 0.2% nitrogen followed, achieving reductions of 2.3 log CFU for *B. atrophaeus* and 2.2 log CFU for *B. subtilis* . The study also revealed that the resistance of *B. subtilis* PS832 (wild‐type) was more excellent than that of *B. atrophaeus* , highlighting species‐specific variability in susceptibility to plasma treatment (Wang, Liu, et al. 2024).

### 2.6 Effect of Contact Time

Contact time is a critical parameter in plasma treatment, as the duration of exposure directly influences the sporicidal effect. Studies have investigated the combined effects of input voltage and treatment duration on bacterial inactivation. Findings suggest a direct relationship between treatment time and the effectiveness of bacterial inactivation. Increased plasma exposure time and higher applied voltages synergistically enhance the sporicidal effect, leading to more effective bacterial deactivation (Wei et al. 2024; Ding et al. 2024).

In this study, the efficiency of cold atmospheric plasma (CAP) generated by a dielectric barrier discharge (DBD) device was evaluated against microbial spores from *Bacillus* spp., *Geobacillus* spp., and *Penicillium* spp., with a focus on the inactivation of spores on dry, heat‐, and water‐sensitive surfaces. The study found that exposure to CAP for just 10 s resulted in up to 3 log^10^ cycles of inactivation for *Bacillus coagulans* spores, with an initial decimal reduction time (D1) of 0.1 min. In contrast, *Bacillus subtilis* spores exhibited the highest resistance, with a D1 of 1.4 min. The study revealed that surface energy plays a critical role in spore inactivation, with higher inactivation rates observed for low surface energy and short exposure times. The inactivation of spores was largely attributed to the erosion of the spore coat induced by reactive nitrogen species (RNS) and charged species, which efficiently interact with the spore surface under increasing electric field strength. UV radiation contributed minimally to the inactivation process. Furthermore, the presence of powders, such as starch granules or diatom shells, reduced the efficiency of spore inactivation, with porous materials offering better shielding of the spores. The study suggests that increasing plasma power and employing fluidized bed technology could enhance the effectiveness of CAP for large‐scale decontamination, while acknowledging the limitations imposed by shielding effects in porous materials (Beyrer, Pina‐Perez, et al. 2020; Beyrer, Smeu, et al. 2020).

This study demonstrated that cold plasma can efficiently inactivate spores of *Alicyclobacillus acidoterrestris* , a highly heat‐resistant spoilage bacterium in fruit juices. The results indicated that a maximum reduction of 4.14 log CFU/mL was achieved within only 7 s in saline solution, and 99% inactivation was obtained within 60 s in apple juice under low‐voltage conditions (1.32 kV), with minimal impact on juice quality. A biphasic inactivation pattern was observed, highlighting a rapid initial kill followed by a slower phase. Among the tested variables, treatment time, input power, and gas flow rate significantly influenced the efficacy. Mechanistic investigations revealed that singlet oxygen (O_2_), generated directly by the plasma, played a dominant role in spore inactivation. Cold plasma caused structural and molecular damage to bacterial spores, affecting membrane integrity, DNA, proteins, lipids, and carbohydrates. These findings confirm that effective spore inactivation can be achieved in short treatment durations, particularly within the first few seconds, and is closely linked to the generation of reactive species, especially O_2_ (Ding et al. 2023) (Table 1).

**TABLE 1:** A summary of studies regarding the effective factors in inactivation of spores using cold plasma.

Effective factor	Bacterial spore	Condition	Results
Exposure mode	*B. atrophaeus*	Direct and indirect	Plasma with direct exposure exhibits a more effective sporicidal action compared to indirect plasma exposure (Los et al. 2020; Wang, Yan, et al. 2024)
Input voltage	*B. licheniformis*	70 kV	Optimum deactivation of *B. licheniformis* spores was reported at a voltage of 70 kV (Huang et al. 2023)
Type of gas	*B. atrophaeus* and *B. subtilis*	A combination of three gases: argon, oxygen and nitrogen	The highest inactivation efficiency showed pure argon as carrier gas with a 3.1 log reduction for *B. atrophaeus* and 2.4 log1 for *B. subtilis* after 5 min plasma exposure followed by argon +0.135% vol. oxygen +0.2% vol. nitrogen with 2.3 log reduction for *B. atrophaeus* and 2.2 log for *B. subtilis* . According to the results, the resistance of the *B. subtilis* PS832 (wild‐type) was higher than *B. atrophaeus* (Wang, Liu, et al. 2024)
	*B. subtilis*	Mixture of O_2_, O_2_/Ar, O_2_/H2 and O_2_/Ar/H2, CO_2_, and O_2_/CF4	The combination of O_2_, O_2_/Ar, O_2_/H_2_, and O_2_/Ar/H_2_, along with CO_2_ and O_2_/CF_4_, resulted in only a 2 log reduction in spore inactivation. In contrast, when the feeding gas consisted solely of a mixture of O_2_ and CF_4_, the reduction in spores increased significantly to a 5 log reduction (Hati et al. 2018)
	*B. subtilis*	Air, nitrogen, oxygen, and CO_2_	A diffuse coplanar surface barrier discharge plasma plate operating at 20 kV, 15 kHz, and for a duration of 7 min was utilized with four working gases: air, nitrogen, oxygen, and CO_2_. The most significant deactivation was noted when nitrogen served as the feed gas (Kitsiou et al. 2024)
Power, frequency and time	*B. subtilis* and *B. atrophaeus*	Power:1.2 kW Frequency:2.45 GHz Treatment time: 30 min	After using microwave‐driven plasma to treat pepper for 30 min, *S. enterica* , *B. subtilis* , and *B. atrophaeus* spores were reduced by 4.1, 2.4, and 2.8 log (Wang, Liu, et al. 2024)
	*B. cereus, B. atrophaeus*, and *G. stearothermophilus*	5, 10, 15, and 20 min	The same level of inactivation was noted for all three strains after the initial 15 min of processing. However, after 20 min, *B. atrophaeus* exhibited the highest inactivation at 4.9 log reduction, followed by *G. stearothermophilus* with a 4.2 log reduction, while *B. cereus* , the most resistant strain, showed a 3.7 log reduction (Valdez‐Narváez et al. 2024)
The structure of spore	*B. cereus*	Protective effect of the inner membrane, cell wall, cortex, outer membrane, and outer shell from inside to outside	N_2_‐CP free radicals directly attack the outer coat of *B. cereus* spores, disruption of the spore's external structure, loss of integrity, leakage from the cytoplasm, and death (Valdez‐Narváez et al. 2024)
Treatment environment	*B. atrophaeus*	Solid and liquid environments	In model systems involving wheat, barley, distilled water, and rubber, the inactivation process proved to be more difficult on the rubber surface compared to liquid media, primarily because of the cells' adherence to the surface.
			Additionally, wheat and barley offered a certain level of protection against reactive species. (Los et al. 2020)
Environmental parameters	*C. difficile*	Matrix (size and structure), humidity (or water activity), and organic matter	After 10 min, some of the spores were still alive, since the spores were protected by the organic matter that had been added, making them more resistant to the cold plasma (Das et al. 2024)

## 3 Advantages and Limitations

Cold plasma offers several advantages over conventional thermal food processing methods. It requires less energy, has minimal effects on food quality, and operates residue‐free without the need for water. Additionally, cold plasma does not rely on vacuum, heat, pressure, or suction, making it an economical and eco‐friendly method suitable for treating and disinfecting large surface areas (Abbaspour et al. 2024; Khoshkalam Pour et al. 2022; Bermudez‐Aguirre 2020). While many studies have utilized helium, argon, or their mixtures with air as carrier gases, the prevailing trend is to employ ambient air due to its ability to generate highly reactive oxidative free radicals during plasma treatment, which enhances microbial inactivation efficacy (Sarangapani et al. 2018).

Despite its numerous advantages, cold plasma technology has certain limitations. The initial investment costs for plasma equipment, the necessity for specific safety measures, and the requirement for skilled personnel pose significant challenges to its adoption. Additionally, some drawbacks have been observed in specific applications. For instance, lipid oxidation in fish and the degradation of oligosaccharides in juices are notable limitations of plasma treatment, as they can compromise these products' nutritional and sensory qualities.

## 4 Mechanism of the Plasma Effect on Microbial Cells

The inactivation of bacteria by cold plasma is primarily attributed to cell wall breakdown, as Samandeep Kaur et al. (2024) reported. Reactive oxygen species (ROS) generated during plasma treatment oxidize lipids and amino acids in the cell membrane, leading to weakened membranes and significant physical changes. These reactive species can penetrate the cytoplasm, disrupting internal organelles and altering biological activity, ultimately resulting in bacterial inactivation (Kaur et al. 2024).

Cold plasma reactive species target peptidoglycans and lipopolysaccharides in the bacterial cell wall, breaking C‐O, C‐N, and C‐C bonds and compromising the cell wall's structural integrity. This destruction leads to cell death (Roh et al. 2020). Reactive nitrogen species (RNS) produced during plasma treatment contribute to microbial inactivation through pH reduction. RNS hydrolysis and converting free radicals and ozone into acids by altering aldehydes in food decrease the pH of the treated food. Studies have demonstrated that the concentration of free radicals—and consequently the reduction in pH—intensifies with longer treatment durations and higher input power, further enhancing microbial inactivation (Ozen et al. 2022; Lee et al. 2024).

The susceptibility of different bacterial species to plasma treatment has been examined by Venetia Samioti et al. (2024), who investigated the effects of plasma on 3 gram‐positive bacteria ( *B. subtilis* , *Staphylococcus epidermidis* , and *Kocuria carniphila* ) and 3 gram‐negative bacteria ( *Enterobacter cloacae* , *Pseudomonas libanensis* , and *Pseudomonas aeruginosa* ). The study revealed that gram‐positive bacteria generally exhibit greater resistance to plasma treatment than gram‐negative bacteria. For example, *B. subtilis* , with a thick cell wall measuring 55.4 nm, exhibited the highest resistance (< 1 log CFU reduction), while *Pseudomonas aeruginosa* , with a thinner cell wall of 2.4 nm, was completely inactivated.

However, distinguishing between the resistance of gram‐positive and gram‐negative bacteria remains complex due to variations in plasma generator types, food matrices, bacterial species, and other influencing factors. A comprehensive evaluation of all adequate conditions and variables is essential to achieve reliable and accurate conclusions regarding bacterial resistance to cold plasma (Samioti et al. 2024).

### 4.1 Effects of Process Variables on Microbial Cells

Various process variables, including voltage, current, frequency, gas type, flow rate, and electrode distance, influence cold plasma's efficacy in microbial inactivation. These parameters are critical in optimizing plasma sterilization and achieving efficient microbial inactivation. Cold plasma technology, initially developed in the 1960s for sterilizing surfaces of medical instruments and packaging materials, has since been adapted for food preservation and microbial control (Sruthi et al. 2022; Ucar et al. 2021). This technology has distinct advantages over traditional methods, such as reducing microbial load while maintaining food quality and preserving its nutritional value.

The equipment settings, including input power, gas composition, and treatment duration, significantly influence the effectiveness of cold plasma treatment. For example, Wang, Liu, et al. (2024) and Wang, Yan, et al. (2024) showed that adjusting these parameters improves the inactivation of bacterial spores. Higher plasma power input (voltage, current, and frequency) generates more energetic electrons, leading to increased production of reactive species and enhanced inactivation efficiency (Liu et al. 2023).

Kulawik et al. (2023) demonstrated this effect when treating *Brochothrix thermosphacta* 38–2 and *Pseudomonas fragi* 38–8 in commercial fish balls. These bacteria were completely inactivated at 22 and 24 kV after 15 and 12 min of pulsed discharge plasma treatment, respectively. However, no significant inactivation occurred at 16 and 18 kV. This emphasizes the importance of using an adequate voltage threshold to achieve effective microbial inactivation.

Similarly, Abdel‐Naeem et al. (2022) reported that ozone concentrations generated by atmospheric DBD plasma increased with higher voltages. For instance, at 55, 65, and 80 kV for 3 min, the ozone concentrations were 220 ± 10, 550 ± 18, and 950 ± 15 ppm, respectively. When chicken fillets were treated with 80 kV for 3, 6, and 9 min, psychrophilic and mesophilic bacterial counts were significantly reduced compared to untreated controls stored at 4°C for 3 days. However, no significant differences in bacterial inactivation were observed between the 3‐, 6‐, and 9‐min treatments, suggesting diminishing returns with prolonged exposure times. A summary of the effective variables on plasma efficacy in germicidal procedures is presented in Table 2.

**TABLE 2:** Different effective variables on plasma process in the germicidal procedure.

Effective factors	Mechanism of action
Reactive oxygen species	Oxidizing lipids and amino acids, membrane weakening, changes in biological activity (Samioti et al. 2024)
Hydrolysis of active nitrogen species	Formation of nitrogenous acids, reduction of pH (Ozen et al. 2022)
Free radicals and ozone	Conversion into acid, affecting aldehydes, decrease in the pH (Samioti et al. 2024)
Treatment time	Extended treatment time causes the accumulation of free radicals (Zhu et al. 2022)
Types of bacteria	Gram positive bacteria are more resistant than gram negative species (Samioti et al. 2024)
Increasing the voltage, current, and frequency	Accumulation of active species resulting higher efficiency of deactivation (Liu et al. 2023)

**FIGURE 1:**
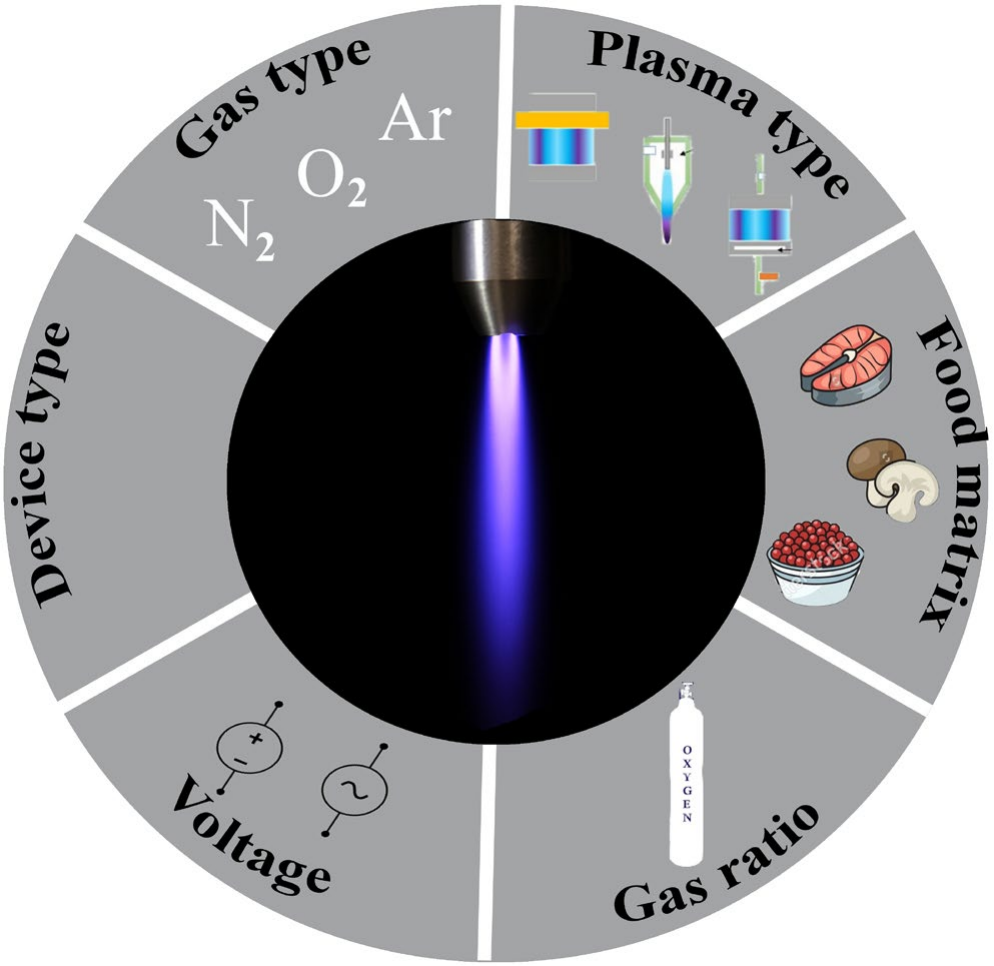
**T**he impact of process variables on the efficiency of cold plasma for the inactivation of spore‐forming microorganisms.

Environmental factors such as food matrix characteristics (size, structure, and water activity), humidity, and organic matter influence plasma efficacy. For instance, organic matter can protect bacterial spores from reactive plasma species, making them more resistant to inactivation. Kumar et al. (2024) reported that *C. difficile* spores treated with cold plasma for 5 min showed partial inactivation, but some spores survived after 10 min, likely due to protection by added organic matter. The choice of gas used in plasma generation significantly affects the chemistry of reactive species, including ionization, UV emission, and the type of radicals formed. Studies have demonstrated varying inactivation rates based on the type of gas used. Wang, Yan, et al. (2024) examined the effects of direct high‐voltage DBD plasma on *B. atrophaeus* spores in sealed packages with three gas compositions. After 30 s of treatment with 70 kV, the results showed a reduction of approximately 1 log CFU with air, < 1 log CFU with 90% N_2_ + 10% O_2_, and 3.8 log CFU with 65% O_2_ + 30% CO_2_ + 5% N_2_. Beyrer et al. (Beyrer, Pina‐Perez, et al. 2020) and (Beyrer, Smeu, et al. 2020) further highlighted the impact of gas type in their study on *B. subtilis* in Spirulina powder. Using micro‐discharge cold atmospheric plasma with a discharge power of 2.2 W, they achieved inactivation rates of 1.63 log CFU with atmospheric gas and 2.12 log CFU with nitrogen after 5 min of treatment. These findings underscore the importance of tailoring the gas composition to specific microbial targets and food matrices. Determining the optimal gas composition and voltage range depends on multiple factors, including the nature of the plasma discharge, operational parameters, substrate characteristics, and the type of microbial contamination. The food matrix is also crucial, as its physical and chemical properties can influence plasma penetration and efficacy. For example, while higher voltage and power inputs generally enhance inactivation, excessive levels may compromise food quality or lead to energy inefficiencies. Figure 1 illustrates the impact of process variables on the efficiency of cold plasma in the inactivation of spore‐forming microorganisms.

## 5 Cold Plasma Application to Spore‐Forming Bacteria

Endospore‐forming bacteria are traditionally classified into two major orders: Bacillales, which includes aerobic, rod‐shaped bacteria, and Clostridiales, comprising strictly anaerobic bacteria. The genera *Bacillus* and *Clostridium* are prototypical representatives of these classifications. Food spoilage is closely associated with the germination and growth of spores, which are critical in determining the stability of heat‐treated foods. Spoilage often manifests as changes in odor, texture, pH, and gas production, which are influenced by the specific microbial species and the composition of the food matrix (Pahalagedara et al. 2024).

Spores are metabolically inactive, with no endogenous or exogenous metabolic processes. This dormancy is primarily attributed to the low water content in the spore core, which restricts protein mobility and enzymatic activity. This desiccated state, combined with a multilayered protective structure, makes spores exceptionally resistant to extreme environmental conditions, including heat, desiccation, and chemical agents.

Certain spore‐forming bacteria, particularly *Bacillus* and *Clostridium* species, are known for producing toxins, leading to foodborne illnesses and spoilage. For example, *Clostridium botulinum* produces botulinum toxin, one of the most potent neurotoxins, while *Bacillus cereus* can cause food poisoning through enterotoxin and emetic toxin production. These toxins pose significant risks to human and animal health and contribute to food spoilage, particularly in improperly stored or treated food products.

**FIGURE 2:**
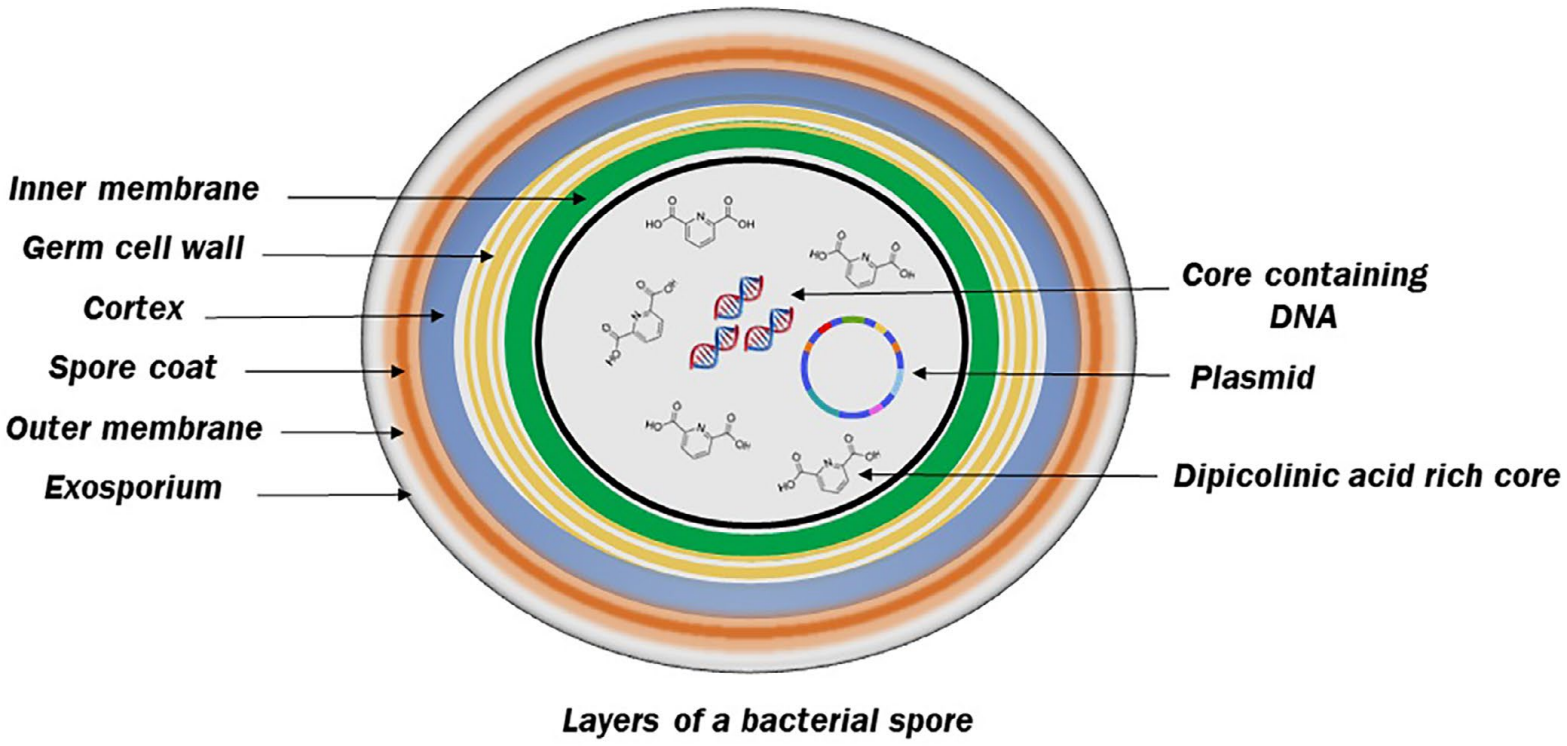
The structure of bacterial spores and the different protective layers against environmental factors.

Given the critical challenges spore‐forming bacteria pose, cold plasma technology has emerged as a promising tool for spore inactivation and reducing contamination in food systems. Cold plasma has demonstrated the ability to inactivate spores by disrupting their protective layers and internal components, leading to structural damage and loss of viability. Figure 2 illustrates the different protecting layers in the structure of the spore. This non‐thermal technology is particularly advantageous for food preservation, as it reduces microbial contamination without compromising food quality.

### 5.1 *Bacillus* spp.

*Bacillus* and *Clostridium* spores under specific plasma conditions. These include adjustments in gas composition, power input, and treatment duration, all of which influence the generation of ROS and RNS responsible for spore destruction. The *Bacillus* genus encompasses a large, heterogeneous group of rod‐shaped, Gram‐positive, facultatively anaerobic, and endospore‐forming bacteria commonly found in soil and water (B. Zhang et al. 2022).

#### 5.1.1 Pathogenic *Bacillus* Species

Pathogenic species within the *Bacillus* genus include *Bacillus anthracis* (the causative agent of anthrax, a zoonotic disease), *Bacillus cereus* (associated with foodborne illnesses and opportunistic infections), and *Bacillus thuringiensis* (pathogenic to invertebrates). Other species, such as *Bacillus licheniformis* , have also been implicated in foodborne illnesses and infections in humans and animals. The endospores of *B. anthracis* are the infectious agents responsible for anthrax, one of the most resilient and potentially deadly zoonotic diseases. *B. anthracis* is unique among *Bacillus* species for its ability to cause epidemic outbreaks in humans and other mammals (Cote et al. 2024). The anthrax toxin is composed of three proteins—protective antigen (PA), edema factor (EF), and lethal factor (LF)—which together play a critical role in disease manifestation.

The mode of entry of *B. anthracis* spores determines the clinical presentation of anthrax. Approximately, 95% of human anthrax cases are cutaneous, involving entry through skin lesions. The remaining 5% are inhalation anthrax (respiratory) or gastrointestinal anthrax resulting from ingestion.

*Bacillus cereus* is a well‐known cause of foodborne illnesses, presenting in two distinct syndromes: diarrheal and emetic.

- *Diarrheal Syndrome*: This syndrome has an incubation period of 8–16 h and lasts 12–24 h. Symptoms include abdominal pain, profuse watery diarrhea, and rectal tenesmus. Nausea and vomiting are less common. The symptoms closely resemble those caused by *Clostridium perfringens* . Foods implicated include meat, vegetables, sauces, pasta, desserts, and dairy products.
- *Emetic Syndrome*: This syndrome has a shorter incubation period of 0.5–5 h and is characterized by nausea and vomiting, with symptoms lasting 6–24 h. The most commonly implicated food is boiled rice stored at room temperature for extended periods before reheating, although dairy products and other starchy foods may also be involved.

Both syndromes occur when spores survive thermal processing and germinate under improper refrigeration, producing toxins that result in illness. *Bacillus thuringiensis* is pathogenic to invertebrates and is widely used as a biopesticide due to its production of insecticidal toxins. However, some strains have been sporadically associated with foodborne illnesses in humans. Additionally, *B. licheniformis* has been implicated in food poisoning and opportunistic infections in humans and animals. Pathogenic *Bacillus* species in the food supply chain significantly challenge food safety. Spores that survive heat treatments can germinate and proliferate, leading to contamination and spoilage. These issues highlight the importance of adopting advanced technologies, such as cold plasma, to mitigate the risks associated with *Bacillus* contamination, inactivate spores, and prevent toxin production.

#### 5.1.2 Bacillus subtilis

*Bacillus subtilis is* commonly present in various foods, particularly in dishes that combine meat or fish with cereal‐based components such as bread, pastries, rice, or stuffing. Numerous reports have identified *B. subtilis* as a causative agent of foodborne illnesses, with vomiting being the most frequently observed symptom. Diarrhea is also commonly associated with these cases.

Symptoms generally begin within short timeframes, with a median onset of 2.5 h. In approximately one‐third of reported cases, symptoms appear within 60 min or less, with an overall range of 10 to 14 h (Zhang et al. 2025). Vomiting is the predominant symptom, occurring in 80% of cases, while diarrhea occurs in 49%. Additionally, about 27% of individuals experience abdominal cramps and pain. In some cases (approximately 10%), other symptoms such as headaches, flushing sensations, or sweating have also been reported.

The rapid onset of symptoms following consumption of contaminated food highlights the significance of *B. subtilis* in food safety. Its prevalence in food matrices and its potential to cause illness underscore the necessity for proper food handling, storage, and preparation practices to mitigate the risks associated with this bacterium.

#### 5.1.3 Bacillus licheniformis

*Bacillus licheniformis* has been associated with foodborne illnesses characterized by the onset of symptoms within 2–14 h (median 8 h). Diarrhea is the predominant symptom, reported in 92% of cases, followed by vomiting (54%) and abdominal cramps or pain (46%). The duration of illness typically ranges from 6 to 24 h. This bacterium has been found in various foods, including ice cream, desserts, meat pies, and sandwiches (Dodd 2017).

#### 5.1.4 Bacillus pumilus

*Bacillus pumilus* is a Gram‐positive, spore‐forming bacterium frequently linked to foodborne illnesses caused by intoxication. This bacterium can proliferate effectively at low temperatures, around 10°C–15°C, and produce significant amounts of toxic metabolites.

The growing consumer demand for fresh‐like food products with high safety standards has driven increased interest in developing non‐thermal methods to control pathogenic microorganisms. These methods, including cold plasma technology, are effective in inactivating *Bacillus* spores and preventing their germination and growth. Recent studies have explored using cold plasma as a non‐thermal inactivation technique to target *Bacillus* and *Clostridium* spores in various food products. Table 3 summarizes key findings regarding the application of cold plasma for spore inactivation, the types of susceptible foods, and the associated symptoms of intoxication (Qi et al. 2024).

**TABLE 3:** A brief review of key findings regarding the application of cold plasma for spore inactivation, the susceptible foods, and the associated symptoms.

Bacterial spp.	Associated foods	Related diseases and symptoms
*B. cereus*	**Emetic syndrome**: Pasta, rice dishes, beef, poultry, milk, pudding, vanilla, sauce, infant formulas **Diarrheal syndrome**: Meat, fish, soups, dairy products, vegetables such as corn, cornstarch, and mashed potatoes	Food poisoning, fulminant bacteremia, meningitis, brain abscesses, endophthalmitis, pneumonia, and gas gangrene‐like cutaneous infections. Vomiting and diarrhea is induced by cereulide and diarrheagenic toxins, respectively (Bhunia 2018)
*B. subtilis*	Meat, fish, bread, rice, seasoning, vegetables, pastry products, sandwiches, pizzas	Vomiting, diarrhea, abdominal cramps/pain, headaches, flushing sensations or sweating as additional symptoms (T. Zhang et al. 2025)
*B. licheniformis*	Ice cream, desserts, meat, pies, sandwiches, meat, bread, pastry products, chicken	Diarrhea, vomiting, abdominal cramps/pain (Soares et al. 2023)
*B. pumilus*	Reheated rice, meat products, sandwiches, canned tomato juice	Vomiting, diarrhea, dizziness, acute gastroenteritis, headache, chills, and elevated heart rate (From et al. 2007)
*C. botulinum*	Salted ham, canned foods, home‐canned vegetables, beans, peppers, carrots, corn, asparagus, potatoes, bamboo, shoots, fish, yogurt, cream cheese, jarred peanuts, blood sausage	Botulism, infant botulism, wound botulism, flaccid paralysis (Bhunia 2018).
*C. perfrigens*	Meat, beef, poultry products, sauces, and stews	Gas gangrene, food poisoning, clostridial myonecrosis, enteritis in animals and humans, enterotoxemia in sheep (struck), and pigbel in humans (Bhunia 2018)
*C. difficile*	Canned salmon, vegetables and seafood	Both gut colonizer and a cause of diarrhea in cattle and poultry. Antibiotic‐associated membranous colitis (diarrhea) in humans (Bhunia 2018)

Research on spore inactivation using cold plasma has demonstrated several potential mechanisms by which chemical species and ultraviolet radiation contribute to the disruption of spores. *Bacillus* spores exhibit remarkable resistance to preservation techniques due to their structural complexity and chemical composition. The spore is encapsulated by a multilayered coat, predominantly composed of proteins (80%) and a smaller proportion of carbohydrates (6%), which acts as a protective barrier against various physical and chemical agents (Prakash et al. 2023).

The primary mechanism of spore inactivation involves the impact of ultraviolet (UV) radiation on DNA, combined with intrinsic photodesorption and the etching of organic molecules on the spore surface (Wang, Liu, et al. 2024). Additionally, reactive species generated by cold plasma have been shown to cause shrinkage, membrane rupture, and leakage of cytoplasmic contents, ultimately leading to spore death (Zhu et al. 2022).

In a study by Wang, Liu, et al. (2024), the effect of four working gases—air, nitrogen, oxygen, and CO_2_—on *B. subtilis* spore inactivation was examined using a diffuse coplanar surface barrier discharge plasma plate (20 kV, 15 kHz) over a 7‐min treatment period. Nitrogen produced the highest level of spore deactivation, underscoring the importance of feed gas composition in optimizing plasma efficacy.

Senguler et al. (2024) conducted an illustrative study on *Bacillus cereus* spores inoculated in onion powder, comparing the effects of low‐microwave density plasma (170 mW/m^2^), high‐microwave density plasma (250 mW/m^2^), and cold plasma processing (400 W, helium, 40 min). High‐microwave density plasma reduced only 2.1 log CFU, while longer processing times enhanced spore inactivation. Cold plasma was more effective due to generating a higher concentration of reactive chemical species and their sustained interaction with spores. However, significant alterations in the volatile profile of the onion powder were observed, indicating that the treatment conditions can influence food quality.

### 5.2 *Clostridium* spp.

The *Clostridium* genus comprises diverse bacteria with varying phenotypes, including psychrophilic, thermophilic, and acidophilic species. These rod‐shaped, anaerobic, endospore‐forming bacteria are oxygen‐tolerant and inhabit diverse ecological niches such as soil, water, and living organisms. Under favorable conditions, the endospores germinate into fully active vegetative cells. The spores are spherical or oval in shape and are highly resistant to chemicals, heat, and desiccation, enabling their survival in adverse environments. The genus includes approximately 235 species and subspecies, many of which produce toxins with harmful effects, causing diseases in plants, animals, and humans (Zeiller et al. 2015).

The heat resistance of clostridial spores varies by species, but the spores of *C. botulinum* and *C. perfringens* are particularly concerning due to their pathogenicity and association with foodborne illnesses. Spores may survive and germinate during standard cooking procedures when nutritional and environmental conditions become favorable, leading to vegetative growth. This ability to form highly resistant spores makes *C. botulinum* a “target organism” in developing time–temperature–heat treatment protocols for canned foods. Proper thermal processing is essential to prevent these products' spore germination and toxin production.

Several species within the *Clostridium* genus are important in human disease due to their production of potent toxins. *C. botulinum* produces botulinum toxin, which causes botulism, a potentially fatal neuroparalytic condition. *C. perfringens* is associated with foodborne gastroenteritis and gas gangrene. *Clostridium tetani* produces tetanospasmin, a neurotoxin responsible for tetanus. *C. difficile* is linked to pseudomembranous colitis, particularly in antibiotic therapy patients. Diseases caused by these species are primarily the result of toxin production, highlighting the critical importance of controlling these bacteria in food systems through proper inactivation techniques (Dürre 2021).

#### 5.2.1 Clostridium botulinum

Consumption of food contaminated with *C. botulinum* neurotoxin can lead to botulism, a severe and potentially fatal form of food poisoning. *C. botulinum* was first isolated from salted ham in 1895 by E. van Ermengen and was initially named *B. botulinus* (derived from the Latin word “botulus,” meaning sausage). Most cases of foodborne botulism are caused by *C. botulinum* group I (proteolytic) and group II (non‐proteolytic), while group III is associated with botulism in birds and animals (Rawson et al. 2023).

*Clostridium botulinum* produces seven recognized serotypes (A through G), all of which share a common structural feature: a disulfide bridge connecting their neurotoxins' heavy and light chains. The heavy chains transport the light chains to the motor neuron cytosol, where they exert their toxic effects. Neurological symptoms of botulism typically begin with blurred vision and can progress to rigid paralysis of respiratory muscles, which may result in death if untreated (Cersosimo et al. 2024).

The types of *C. botulinum* are also distinguished by their proteolytic properties. Proteolytic strains produce enzymes that break down proteins, resulting in a musty, unpleasant odor in contaminated food and deterring consumption. However, all strains are saccharolytic, capable of fermenting glucose to generate energy while producing acids and gases as byproducts. Notably, approximately 72% of botulism outbreaks have been linked to homemade canned foods and vegetables. Foodborne botulism occurs when preformed botulinum neurotoxin is ingested, with as little as 30 ng of toxin sufficient to cause illness or death (Meurens et al. 2023).

Recent outbreaks of foodborne botulism linked to proteolytic *C. botulinum* have been associated primarily with improperly canned foods, while fish products, such as smoked, dried, or salted fish, have been the most common source of outbreaks caused by non‐proteolytic *C. botulinum* . In Europe, outbreaks are frequently linked to type B neurotoxins produced by non‐proteolytic strains of *C. botulinum* type B (Pahalagedara et al. 2024).

#### 5.2.2 Clostridium perfringens

*Clostridium perfringens* is a Gram‐positive, spore‐forming, anaerobic, and nonmotile rod‐shaped bacterium that forms large, regular, round, slightly opaque, and shiny colonies on agar plates. The bacterium grows optimally at temperatures between 37°C and 45°C but is capable of growth within a broader range of 6°C to 50°C. Although *C. perfringens* is found in diverse environments, it is predominantly located in the intestines of healthy and diseased animals (Shamshirgaran and Golchin 2025). The symptoms of *C. perfringens* infections vary depending on the types of toxins produced. Infections can manifest as fever, abdominal pain, diarrhea, and, less commonly, vomiting. Additionally, *C. perfringens* can cause gastrointestinal disorders, liver and kidney damage, dermatitis, and gas gangrene. Among the *Clostridium* genus, this bacterium is notable for being the most prolific producer of toxins, synthesizing over 20 toxins and enzymes (Yadav et al. 2022).

The virulence of *C. perfringens* is attributed to its ability to produce toxins grouped into four categories: membrane‐damaging enzymes, pore‐forming toxins, intracellular toxins, and hydrolytic enzymes. In developed nations, *C. perfringens* is the second most common cause of foodborne intoxication (Rood et al. 2018). The bacterium's growth heavily depends on the availability of amino acids and vitamins commonly found in meat products. As a result, the primary food vehicles for *C. perfringens* food poisoning include meat (notably beef and poultry) and meat‐based dishes such as gravies, stews, and Mexican foods.

Foodborne illnesses caused by *C. perfringens* are typically associated with improper food preparation practices, particularly temperature abuse during cooking, cooling, or storage. Such conditions allow spores to survive and germinate, leading to vegetative growth and toxin production in food (Yang and Scharff 2024).

#### 5.2.3 Clostridium tetani

*Clostridium tetani* is a slender, Gram‐positive, rod‐shaped, motile, nonencapsulated, and obligately anaerobic bacillus in vegetative and sporulated forms. It is primarily known as the causative agent of tetanus. The sporulated form is characterized by terminal spores that give it a distinct drumstick‐like microscopic appearance. This feature contributes to the spores' resistance to heat and many antiseptics, which helps preserve the organism's chemical structure. Although *C. tetani* can be found in various environments, its natural habitats include the intestinal tracts of animals, soil, and dust (Kiron et al. 2024). Under anaerobic conditions, *C. tetani* secretes two toxins: tetanolysin, which causes local tissue damage, and tetanospasmin, which is responsible for the clinical symptoms of tetanus syndrome. The high pathogenicity of tetanospasmin, a potent neurotoxin, underscores the importance of preventive measures such as vaccination to control tetanus outbreaks (Pirazzini et al. 2022).

#### 5.2.4 Clostridium difficile

*Clostridium difficile* is a Gram‐positive, anaerobic, spore‐forming, and toxigenic bacillus widely distributed in the environment. The spores are primarily spread through feces and saliva, and potential reservoirs include asymptomatic carriers, infected patients, contaminated environments, and the intestinal tracts of animals such as canines, felines, porcines, and avians (Okafor et al. 2023). *C. difficile* was first isolated from the stool of a healthy newborn by Hall and O'Toole in 1935 (Shirley et al. 2023), *and* it is now recognized as a significant human pathogen. It is associated with severe intestinal disease, including pseudomembranous colitis, is a significant cause of nosocomial diarrhea worldwide (Sullivan et al., 1982). *C. difficile* produces two major exotoxins, toxin A (TcdA) and toxin B (TcdB), which are key virulence factors secreted into the colonic environment. These toxins are responsible for disrupting intestinal epithelial cells and the associated pathogenesis. Additionally, a binary toxin known as CDT is present in certain hypervirulent strains and is linked to more severe disease outcomes, although it has not been shown to cause disease independently (López‐Cárdenas et al. 2021).

Traditionally considered a healthcare‐associated infection, *C. difficile infection* (CDI) is increasingly recognized as a potential foodborne pathogen. The transmission of *C. difficile* spores through the food chain has gained attention due to the detection of genetically similar strains in animals, humans, and retail food products. The germination of *C. difficile* spores in the gastrointestinal tract requires bile salts and an anaerobic environment, enabling the transformation of spores into vegetative cells (Remize and De Santis 2025). Research has focused mainly on slaughterhouse animals, carcasses, and retail meats as potential sources of contamination. However, spores have also been detected in other food sources, including vegetables and seafood. This expanding evidence has prompted heightened surveillance and research into the role of *C. difficile* as a potential foodborne agent.

## 6 Comparison of Cold Plasma vs. Other Non‐Thermal Technologies

Cold plasma treatment provides notable advantages over traditional food preservation methods, such as high hydrostatic pressure (HHP), pulsed electric field (PEF), radiation, and conventional high‐temperature techniques, including pasteurization and sterilization. Operating at ambient temperatures, it effectively inactivates microbes in sensitive food matrices without changing product quality. While pasteurization reduces microbial loads, it often fails to fully inactivate resilient spores (Nikmaram and Keener 2022). However, cold plasma generates reactive species that disrupt spore structures more effectively than radiation methods, which can cause collateral damage and require longer exposure times.

Furthermore, cold plasma is a more adaptable alternative to high hydrostatic pressure, which necessitates complex equipment and is limited by the structure and composition of the food (Kulawik et al. 2023). It can be seamlessly integrated into existing food production processes, enhancing operational efficiency. Compared to pulsed electric field technology, cold plasma's ability to produce various reactive species allows it to function effectively in diverse environments (Dalvi‐Isfahan and Mahmoodi‐Eshkaftaki 2024). Overall, cold plasma technology stands out as a versatile and efficient solution for microbial spore inactivation, particularly in applications requiring gentle treatment that preserves food integrity.

## 7 Conclusions and Future Prospectives

The presence of spores in food presents a significant challenge to food safety due to their exceptional resistance to environmental stresses. The sporulation process is intricately regulated by genetic and environmental factors within vegetative cells, enabling spores to endure thermal and non‐thermal food processing methods. The biochemical mechanisms, particularly the interactions between reactive plasma species and the structural components of spores, such as their proteinaceous coat and DNA, are among the main effective parameters. Future investigations should focus on the impact of various treatment mediums, such as food matrices and environmental conditions, on the efficiency of spore inactivation and the specific conditions required for different types of spores originating from diverse food sources. Additionally, there is a need to explore new effective germination factors and the potential combinations of existing ones. The increasing interest in non‐thermal preservation methods, spurred by consumer demand for minimally processed foods devoid of chemical preservatives, emphasizes the importance of innovative approaches like cold plasma technology. The introduction of novel ingredients may lead to the emergence of new spoilage organisms and spore formers that have not been previously considered in various food products. Such research endeavors will aid in refining cold plasma applications and affirming its efficacy as a holistic strategy for reducing foodborne spore contamination. In summary, cold plasma technology offers significant advantages regarding food safety, quality, and enhancement of nutritional value. As advancements in this technology progress, it is likely to play a crucial role in transforming the food industry and addressing the changing needs and preferences of consumers globally.

## Author Contributions

**Shiva Ezzati:** data curation (equal), formal analysis (equal), validation (equal), visualization (equal), writing – original draft (equal). **Hossein Ahangari:** methodology (equal), project administration (equal), visualization (equal), writing – original draft (equal), writing – review and editing (equal). **Mohadeseh Mohammadian:** formal analysis (equal), methodology (equal), writing – original draft (equal). **Ali Khoshkalampour:** data curation (equal), investigation (equal), software (equal). **Ehsan Moghaddas Kia:** project administration (equal), resources (equal), supervision (equal), visualization (equal), writing – original draft (equal), writing – review and editing (equal). **Zahra Ghasempour:** formal analysis (equal), project administration (equal), software (equal), supervision (equal), validation (equal), visualization (equal), writing – review and editing (equal).

## Ethics Statement

The protocol of this study was approved by the Ethics Committee of Maragheh University of Medical Sciences (Ethical cod: IR.MARAGHEHPHC.REC.1404.007).

## Conflicts of Interest

The authors declare no conflicts of interest.

## Data Availability

Data sharing is not applicable to this article as no datasets were generated or analyzed during the current study.
